# Hospital length of stay and readmission after elective surgery: a comparison of current and former smokers with non-smokers

**DOI:** 10.1186/s12913-024-10566-3

**Published:** 2024-01-17

**Authors:** Gina Arena, Craig Cumming, Natalia Lizama, Hamish Mace, David B. Preen

**Affiliations:** 1 School of Population and Global Health M431, The University of Western Australia, 35 Stirling Highway, Crawley, Western Australia 6009 Australia; 2 Cancer Council Western Australia, Subiaco, Western Australia Australia; 3 Curtin University, Bentley, Western Australia Australia; 4 Division of Emergency Medicine, The University of Western Australia, Perth, Western Australia Australia; 5 Department of Anaesthesia and Pain Medicine, Fiona Stanley Hospital, Murdoch, Western Australia Australia

**Keywords:** Cessation, Prevention, Public policy, Nicotine, Smoking caused disease

## Abstract

**Background:**

The purpose of this study was to investigate differences between non-smokers, ex-smokers and current smokers in hospital length of stay (LOS), readmission (seven and 28 days) and cost of readmission for patients admitted for elective surgery.

**Methods:**

A retrospective cohort study of administrative inpatient data from 24, 818 patients admitted to seven metropolitan hospitals in Western Australia between 1 July 2016 and 30 June 2019 for multiday elective surgery was conducted. Data included smoking status, LOS, procedure type, age, sex and Indigenous status. LOS for smoking status was compared using multivariable negative binomial regression. Odds of readmission were compared for non-smokers and both ex-smokers and current smokers using separate multivariable logistic regression models.

**Results:**

Mean LOS for non-smokers (4.7 days, SD=5.7) was significantly lower than both ex-smokers (6.2 days SD 7.9) and current smokers (6.1 days, SD=8.2). Compared to non-smokers, current smokers and ex-smokers had significantly higher odds of readmission within seven (OR=1.29; 95% CI: 1.13, 1.47, and OR=1.37; 95% CI: 1.19, 1.59, respectively) and 28 days (OR=1.35; 95% CI: 1.23, 1.49, and OR=1.53; 95% CI: 1.39, 1.69, respectively) of discharge. The cost of readmission for seven and 28-day readmission was significantly higher for current smokers compared to non-smokers (RR=1.52; 95% CI: 1.1.6, 2.0; RR=1.39; 95% CI: 1.18, 1.65, respectively).

**Conclusion:**

Among patients admitted for elective surgery, hospital LOS, readmission risk and readmission costs were all higher for smokers compared with non-smokers. The findings indicate that provision of smoking cessation treatment for adults undergoing elective surgery is likely to produce multiple benefits.

## Introduction

Smoking is responsible for a large burden of disease and deaths globally [1] and is associated with health problems including cardiovascular disease and cancer [2]. Despite being one of the leading causes of preventable disease and death, 10.7% of Australians continued to smoke daily in 2020/21 [3]. Understanding the impact of smoking on health service use is critical to inform policies aimed at implementing smoking cessation programs and improving health.

Tobacco-induced damage to organs and the health effects of smoking and the associated costs have been established in the literature [4]. This damage can be partially reversed with smoking cessation [5]. Smokers are over-represented in hospital admissions for a range of chronic health conditions and -their health service usage is disproportionate [5]. These admissions linked to individuals with risk factors, such as smoking, increases the risk associated with hospitalisations for surgical procedures [6]. Tobacco smoke adversely impacts the tissue microenvironment; carbon monoxide bound to haemoglobin (carboxyhaemoglobin) impairs oxygen delivery, while nicotine acts as a potent vasoconstrictor, reducing circulation to healing tissues. Following surgery, smokers demonstrate poor wound healing, and have an elevated incidence of post-operative infections including surgical-site and respiratory infections [7]. Research has also shown that smokers with chronic diseases have higher rates of hospital readmissions than non-smokers [8, 9].

Tangible and intangible costs associated with smoking continue to rise in Australia and were estimated at $137 billion in 2015/16 [10]. These costs include premature mortality, workplace costs and health care costs. Tobacco cessation programs can reduce these costs, increase quality of life and positively impact the wider community [11]. Despite considerable efforts taken to reduce smoking, the adverse impacts and costs continue [10].The World Health Organization Framework Convention on Tobacco Control recommends the provision of further evidence to support tobacco cessation alongside comprehensive tobacco control measures preventing the uptake of smoking [5, 12]. The existing body of evidence is limited regarding the impact of smoking on key health care metrics, notably the length of stay (LOS) following surgery and the occurrence of surgical readmissions, with particular emphasis on elective procedures. Elective procedures add a layer of specificity as they are planned by the patient. Studies addressing the impact of smoking on LOS exist, however they focus on specific surgeries and complications (i.e., non-elective skull based surgery) and have focused on single institutes and single surgeon studies and have not been population based [13, 14]. This study aims to investigate the differences between non-smokers against ex-smokers and current smokers in hospital LOS, readmission within seven and 28 days post-discharge and cost of readmission in patients undergoing elective surgery.

## Methods

### Study design

A retrospective cohort study was conducted of administrative inpatient data comprising patients admitted to hospital for elective surgery. Adult (≥18 years) patients whose admission was at least two days in duration were included. This study focused on admissions for elective surgery lasting at least two days, in order to remove admissions for minor procedures performed in day surgery, which are recorded as single-day admissions.

### Data collection

Unit-record data were obtained for all hospital separations at seven public metropolitan hospitals in Perth, Western Australia, between 1 July 2016 and 30 June 2019. Data were de-identified, with a scrambled version of individual participants' unit medical record number (UMRN) that was used to record their admissions. Demographic variables included patient sex, age and Indigenous status. Hospital service data included dates of admission and discharge, principal and secondary diagnoses coded using International Statistical Classification of Diseases, 10^th^ revision, Australian Modification (ICD-10-AM) [15] codes (up to 104). Principal and all secondary procedures were coded using Australian Classification of Health Interventions (ACHI) [16] codes, elective procedure flag, and Australian-defined Diagnostic Related Groups version 8 (AR-DRG) [17] codes.

The Australian Institute of Health and Welfare's National Hospital Morbidity Database [18] was used to identify the AR-DRG codes that corresponded with surgical procedures. The AR-DRG codes from the inpatient data were used with the available elective procedure flag to determine admissions of interest. AR-DRG codes is a classification system that groups patient numbers and types to the resources required in treatment. They are then used, in an algorithm, to calculate public hospital funding on an activity basis [19]. We obtained data on the total cost for each admission of interest which was calculated using AR-DRG codes.

### Exposure measure

Smoking status was ascertained using ICD-10-AM codes Z72.0 and F17 for current smokers, and Z86.43 for ex-smokers. Using each individual's scrambled hospital identifier, the data were interrogated for smoking status consistency throughout the study period. Smoking status was categorised as ‘non-smoker’ (i.e., never smoked), ‘ex-smoker’ or ‘current smoker’.

A total of 56,539 (8.7%) admissions had no smoking status recorded. However, in previous admissions these patients had their smoking status documented. They were then categorised based on the most harmful level of smoking status during the study period (non-smoker being least harmful and current smoker being most harmful). This approach was used because the majority of smokers who are able to quit smoking successfully take multiple attempts [20]. Recent published estimates suggest that the average number of attempts to quit smoking successfully (with cessation lasting at least a year) is 6.1, with a maximum of 142 depending on the statistical approach used [20]. Based on this evidence, the chance that an individual recorded in the data as a current smoker at some stage during the three-year study period successfully quit and became a former smoker in this timeframe was assumed to be low.

### Outcome measures

The primary outcomes of the study were LOS and readmission within seven and 28 days of discharge and cost of readmission. LOS was calculated using the admission and discharge dates for each admission. The principal and secondary AR-DRG codes were used to identify patients with an elective procedure flag. Readmission at seven and 28 days post-discharge were flagged by sorting admissions by hospital identifier and admission date, and calculating the number of days between an individual being discharged from a multiday elective surgery admission and subsequent readmission (with no limitation placed on the readmission type).

### Analysis

Using hospital admission as the unit of analysis, descriptive statistics were calculated for each smoking group: LOS, median age, and proportions of male/female and Indigenous patients. The number of multiday elective surgery admissions was also calculated for each participant. Crude comparative tests were initially performed to examine differences across the smoking groups using a non-parametric equality of medians test (due to age data being skewed) for median age of the patient at admission, and chi-square tests for the proportion of females.

The distribution of LOS was positively skewed, so multivariable negative binomial regression was used to investigate differences in hospital days admitted for multiday elective surgery between the smoking groups. As there was a strong independent association between age and LOS, as well as an interaction between age and smoking status when a Wald test was performed, admissions were separated into age groups (<30, 30-39, 40-49, 50-59, 60-69, 70-79, 80+ years). Analyses were stratified by age group, controlling for sex and Indigenous status.

To investigate the association between smoking status and seven-day and 28-day readmission, we used separate multivariable logistic regression models, controlling for age, sex, Indigenous status and LOS of the surgery admission; LOS has previously been found to be positively associated with an increased risk of readmission [21, 22].

Cost data for each readmission were determined from AR-DRG codes. Cost data were positively skewed, therefore separate multivariable negative binomial regression models investigated the relationship between smoking status and cost of readmission for seven-day and 28-day readmission, controlling for age, sex, Indigenous status and LOS. We performed post-hoc multivariable negative binomial regression analysis to compare cost of seven- and 28-day readmission between the ex-smoker and current smoker groups, this analysis excluded the non-smoker group.

All statistical analyses were performed using Stata version 16 [23] and all multivariable regression models used the cluster Huber/White Sandwich Estimator option [24] to adjust for variance estimates for within-cluster correlation that may be caused by multiple observations for some participants. Where LOS was included in a model as a covariate, it was recoded as a binary variable using three days as the cut-off point, resulting in two groups of approximately equal size (51% with LOS of ≤ 3 days vs 49% with LOS > 3 days). This cut-off was the median admission length, and was also consistent with evidence from previous studies on hospital readmission that found this threshold was clinically meaningful [22, 25].

## Results

### Descriptive characteristics

There were records for 650,199 admissions for 333,334 unique patients identified during the study period; of these, 166,919 admissions (25.7%) were for a surgical procedure, with 97,521 (58.4%) of these for elective procedures. A total of 27,107 (27.8%) of the admissions for elective surgery procedures were for at least two days and were included in the analysis, which related to 24,818 unique patients.

Non-smokers (74% female, median age=47) were significantly younger and significantly more likely to be female than ex-smokers (41% female, median age=67; *p*<0.001) and current smokers (46% female, median age=56; *p*<0.001) (Table 1). The mean LOS for each smoking group (not stratified by age) was 6.1 days (SD=8.2) for current smokers, 6.2 days (SD=7.9) for ex-smokers and 4.7 days (SD=5.7) for non-smokers. In pairwise comparisons LOS was significantly lower for non-smokers compared to both ex-smokers and current smokers (*p*<0.001). Following discharge from multiday elective surgery there were 612 (4%) readmissions within seven-days for non-smokers compared to 475 (6%; *p*<0.001) for ex-smokers and 359 (6%; *p*<0.001) for current smokers. For non-smokers, ex-smokers and current smokers, there were 1335 (10%), 1155 (15%; *p*<0.001) and 889 (16%; *p*<0.001) readmissions, respectively, within 28 days of discharge from multiday elective surgery.

**Table 1:** Multiday elective surgery admissions: patient demographics, mean LOS and proportion followed by readmission within seven and 28 days of discharge

	Non-smoker (*n*=13975)	Ex-smoker (*n*=7542)	Current smoker (*n*=5590)	Total (*n*=27107)
Number of unique individuals	13220	6610	4988	24818
Mean (SD) admissions per individual	1.06 (0.28)	1.12 (0.37)^a^	1.12 (0.38)^a^	1.1 (0.3)
Maximum admissions per individual	6	6	11	11
Mean LOS in days (SD)	4.7 (5.7)	6.2 (7.9)^a^	6.1 (8.2)^a^	5.4 (7.0)
Median age (IQR)	47 (31-68)	67 (56-75)^a^	56 (42-66)^a^	58 (36-70)
Female (%)	10347 (74)	3088 (41)^a^	2577 (46)^a^	16012 (59)
Followed by readmission within 7 days (%)	612 (4)	475 (6)^a^	359 (6)^a^	1446 (5)
Followed by readmission within 28 days (%)	1335 (10)	1155 (15)^a^	889 (16)^a^	3305 (12)

^*^ <0.001

T-test used to compare means

Non-parametric equality of medians test used to compare medians

Chi square test used to compare proportions

### Length of stay

In unadjusted analyses, ex-smokers and current smokers had significantly greater LOS than non-smokers for all but the <30 years and 80+ years age groups (Table 2). After adjusting for sex and Indigenous status, ex-smokers and current smokers aged 60-69 years both had significantly greater LOS than non-smokers in the 60-69 (adjusted Rate Ratio (aRR)=1.12; 95%CI: 1.03, 1.21, and aRR=1.20; 95%CI: 1.09, 1.33, respectively) as did ex-smokers and current smokers aged 70-79 years (aRR=1.10; 95%CI: 1.02, 1.18, and aRR=1.24; 95%CI: 1.13, 1.36, respectively) (Table 2). This equates to a 12% longer stay for ex-smokers and 20% longer stay for current smokers in comparison to non-smokers. Among the 30-39 year age groups, LOS was significantly longer for ex-smokers compared to non-smokers (aRR=1.14, 95%CI 1.02, 127), but there were no significant differences between current smokers and non-smokers (Table 2). Conversely, in the 50-59 year age group, current smokers had a greater LOS than non-smokers (aRR=1.13, 95%CI 1.01, 1.26), but there was no significant difference between ex-smokers and non-smokers (Table 2).

**Table 2:** Crude and adjusted rate ratios for length of stay in days, in current and ex-smokers versus non-smokers, by age

Age group in years	Smoker status	RR (95%CI) (crude)	*p*-value	aRR (95%CI) (adjusted)	*p*-value
<30 (*n*=3496)	Ex-smoker	1.05 (0.93, 1.19)	0.401	1.04 (0.93, 1.17)	0.469
	Current smoker	1.10 (1.02, 1.19)	0.019	0.96 (0.88, 1.06)	0.433
30-39 (*n*=4523)	Ex-smoker	1.26 (1.11, 1.44)	<0.001	1.14 (1.02, 1.27)	0.022
	Current smoker	1.30 (1.19, 1.44)	<0.001	1.02 (0.92, 1.13)	0.711
40-49 (*n*=2574)	Ex-smoker	1.23 (1.05, 1.45)	0.009	1.18 (1.02, 1.36)	0.025
	Current smoker	1.19 (1.05, 1.35)	0.006	1.09 (0.96, 1.25)	0.199
50-59 (*n*=3794)	Ex-smoker	1.15 (1.04, 1.28)	0.008	1.08 (0.97, 1.20)	0.160
	Current smoker	1.20 (1.09, 1.33)	<0.001	1.13 (1.01, 1.26)	0.032
60-69 (*n*=5407)	Ex-smoker	1.15 (1.07, 1.24)	<0.001	1.12 (1.03, 1.21)	0.005
	Current smoker	1.22 (1.12, 1.34)	<0.001	1.20 (1.09, 1.33)	<0.001
70-79 (*n*=5022)	Ex-smoker	1.12 (1.04, 1.21)	0.002	1.10 (1.02, 1.18)	0.010
	Current smoker	1.27 (1.15, 1.40)	<0.001	1.24 (1.13, 1.36)	<0.001
80+ (*n*=2291)	Ex-smoker	0.95 (0.86, 1.05)	0.343	0.97 (0.87, 1.07)	0.513
	Current smoker	1.06 (0.87, 1.30)	0.559	1.08 (0.88, 1.33)	0.478

*RR* Crude rate ratio, *aRR* Adjusted rate ratio

Stratified by age group and adjusted for sex and Indigenous status

### Readmission and cost

Across all groups there were 1424 (6%) of participants who had a seven-day readmission during the study period (*n*=620 (5%) non-smokers, *n*=458 (7%) ex-smokers, *n*=346 (7%) current smokers). There were a total of 3253 (13%) participants with a 28-day readmission during the study period (*n*=1331 (10%) non-smokers, *n*=1082 (16%) ex-smokers, *n*=840 (17%) current smokers). After adjusting for covariates, the odds of seven-day readmission after discharge from multiday elective surgery were greater for both ex-smokers (aOR=1.29; 95%CI: 1.13, 1.47) and current smokers (aOR=1.37; 95%CI: 1.19, 1.59) when compared to non-smokers (Table 3). The odds were higher for 28-day readmission with both ex-smokers (aOR=1.35; 95%CI: 1.23, 1.49) and current smokers (aOR=1.53; 95%CI: 1.39, 1.69) at increased odds of readmission compared to non-smokers.

**Table 3:** Relationships of readmissions within seven and 28 days of discharge from multiday elective surgery, adjusted for age, sex, Indigenous status, and LOS (reference group non-smokers)

	7-day readmission				28-day readmission			
	**OR (95% CI)**	**p**	**aOR (95% CI)**	**p**	**OR (95% CI)**	***p*-value**	**aOR (95% CI)**	**p**
Ex-smoker	1.48 (1.31, 1.67)	<0.001	1.29 (1.13, 1.47)	<0.001	1.72 (1.58, 1.87)	<0.001	1.35 (1.23, 1.49)	<0.001
Current smoker	1.50 (1.31, 1.72)	<0.001	1.37 (1.19, 1.59)	<0.001	1.79 (1.63, 1.96)	<0.001	1.53 (1.39, 1.69)	<0.001
Age group (ref <30)								
30-39			0.98 (0.79, 1.22)	0.890			0.94 (0.80, 1.11)	0.485
40-49			0.91 (0.71, 1.17)	0.464			1.33 (1.12, 1.58)	0.001
50-59			1.01 (0.81, 1.26)	0.934			1.31 (1.12, 1.55)	0.001
60-69			1.01 (0.82, 1.25)	0.890			1.35 (1.16, 1.57)	<0.001
70-79			0.95 (0.77, 1.18)	0.667			1.28 (1.09, 1.49)	0.002
>80			1.06 (0.83, 1.36)	0.631			1.50 (1.26, 1.79)	<0.001
Female			0.81 (0.72, 0.92)	0.001			0.78 (0.72, 0.85)	<0.001
Indigenous			0.80 (0.72, 0.92)	0.166			0.90 (0.74, 1.13)	0.390
Admission > 3 days (ref ≤ 3 days)			1.63 (1.46, 1.83)	<0.001			1.65 (1.50, 1.75)	<0.001

Adjusted logistic regression (aOR)

Unadjusted logistic regression (aOR)

When compared to non-smokers the cost of readmissions for current smokers was significantly higher for both seven-day (aRR=1.51; 95%CI: 1.15, 1.98) and 28-day (RR=1.38; 95%CI: 1.16, 1.64) readmissions, with ex-smokers not differing significantly for either readmission outcome (Table 4). Our post-hoc analysis showed a non-significant difference in cost for seven-day readmission between the current smoker and ex-smoker groups (aRR=1.34; 95%CI: 0.99, 1.80), however the costs for 28-day readmission were significantly greater for current smokers compared to ex-smokers (aRR=1.23; 95%CI: 1.04, 1.47).

**Table 4:** Crude and adjusted rate ratios for total cost of admission for readmission within seven and 28 days of discharge from multiday elective surgery between non-smokers and ex- or current smokers

	7-day readmission				28-day readmission			
	RR	p	aRR (95%CI)	p	RR	p	aRR (95%CI)	p
Ex-smoker	1.42 (1.16, 1.74)	0.001	1.17 (0.97, 1.41)	0.107	1.27 (1.10, 1.46)	0.001	1.12 (0.97, 1.30)	0.127
Current smoker	1.71 (1.25, 2.34)	0.001	1.51 (1.15, 1.98)	0.003	1.49 (1.25, 1.78)	<0.001	1.38 (1.16, 1.64)	<0.001
Age group (ref <30)								
30-39			0.70 (0.45, 1.11)	0.130			0.98 (0.69, 1.39)	0.919
40-49			1.11 (0.69, 1.77)	0.674			1.35 (0.95, 1.90)	0.091
50-59			0.97 (0.62, 1.52)	0.909			1.18 (0.87, 1.61)	0.278
60-69			1.23 (0.78, 1.93)	0.375			1.34 (0.99, 1.82)	0.062
70-79			1.35 (0.84, 2.18)	0.213			1.64 (1.20, 2.24)	0.002
>80			1.31 (0.84, 2.04)	0.231			1.25 (0.92, 1.70)	0.160
Female			0.94 (0.77, 1.14)	0.503			0.89 (0.79, 1.02)	0.092
Indigenous			1.17 (0.72, 1.89)	0.528			1.18 (0.88, 1.57)	0.266
Admission > 3 days (ref ≤ 3 days)			1.46 (1.21, 1.77)	<0.001			1.37 (1.20, 1.57)	<0.001

*RR* Crude rate ratio, *aRR* Adjusted rate ratio

Adjusted for age group, sex, Indigenous status, and length of stay during elective surgery admission (reference group non-smokers)

## Discussion

The results of this retrospective cohort study in Western Australia show that of the 24,818 patients admitted for elective surgery procedures for at least 2 days, compared to non-smokers, current smokers and ex-smokers had a significantly greater LOS and significantly higher readmission rates (at least 43% and 60% higher for each smoker group for seven- and 28-day readmission respectively) following elective surgery. Furthermore, compared to the non-smoker group there was a significantly higher cost per hospital readmission in the current smoker group for both seven-day and 28-day readmissions.

Despite considerable national and international progress in tobacco control, health care costs continue to be impacted by smoking-related illness. Although daily smoking prevalence for Australians over 18 years decreased from 20% in 2001 to 11.6% in 2019, smokers are still over-represented in hospital admissions, placing a significant burden on the health care system [26]. Many of the long-term health effects from smoking develop over years or even decades, leading to serious health problems that require medical attention.

Our results show that the cost of readmission was significantly higher for current smokers compared to non-smokers, but not between non-smokers and ex-smokers. We also found a significant greater cost for 28-day readmissions for current smokers compared to ex-smokers which corresponds with other findings related to smokers [9, 27]. As a modifiable risk factor, this finding provides further support for implementing smoking cessation interventions at a hospital level. Smoking cessation leads to improvements in the tissue microenvironment [7], with oxygen delivery improved by a reduction in carboxyhaemoglobin concentration within as little as three days.

Incorporating cessation programs into hospitals or prior to admission provides an opportunity to reach current smokers in an environment where they are likely more conscious of their health than in the community – a so-called teachable moment [28, 29]. Studies investigating smoking cessation commenced as inpatients have reported increased patient interest in the intervention while hospitalised [30]. It is important to consider efforts to integrate essential components, including follow-up support, into the cessation program to ensure the functionality and effectiveness of the service [5]. Rigotti et al., [6] determined that programs commenced in hospitals and continued for at least one month after discharge increased the likelihood of patients being smoke-free by 37% at six to twelve months after discharge. Targeting smokers during their hospital stay is an opportune time given they are often motivated to quit smoking during their admission, coupled with the effectiveness of hospital-initiated smoking interventions [11] and, in many cases, smoke-free hospital grounds. In contrast, van den Broek-Attenburg and Atherly [31] did not find significant short-term changes and described this in relation to limitations in the research methodology. Mullen et al. demonstrated the cost-effectiveness of a hospital-based strategy, estimating the cost per quality-adjusted life year (QALY) gained at $1,386 (CAN) [11]. The incremental cost, in comparison to standard care, for achieving these QALY gains amounted to a modest $5 per patient, constituting just 0.2% of the total expenditure for treating patients with smoking-related cardiovascular disease [11]. When the financial costs are considered alongside the quality of life gains and long-term reduction in morbidity, there is a clear net economic benefit associated with effective smoking interventions. Additionally, the average LOS among current smokers in our study was around six days, which provides sufficient time to commence a cessation intervention which needs to continue after hospital separation for effectiveness of the program [5].

Strengths of our study include the large sample size and the use of administrative health record data. These data provide an opportunity to look at large and diverse populations using methods that are less expensive and time-consuming than other designs. We attempted to elucidate differences between the smoking groups in rates of admissions (original procedure) for specific surgical procedure subgroups (such as cardiothoracic and circulatory system surgeries) or diagnostic categories (such as surgical site infection and cardiovascular disease), but despite our large sample, there were insufficient data to support this examination. To address existing gaps in the literature, future research should consider investigating relationships between specific surgical procedure-types and smoking status and other causative factors behind readmission.

Our method of classifying participants by smoking status and assigning the (likely) very few participants who did quit successfully during the period to the current smoker group may bias the findings towards the null, resulting in conservative estimates being reported here. Future research looking at a cost-benefit analysis from the perspective of hospitals would provide information regarding readmissions associated with smoking. Limitations relating to the complexities of patients and multidisciplinary care can occur. Although likely representative of a first-world health care system, future studies that can include a longer time-period, capture records for day surgery (<24 hours), have a sub-analysis of different surgical specialties, and include additional Australian or international jurisdictions may provide more information on the impact of nationwide cessation programs and the changing demographics of the smoking population. As well, the validity of administrative records recording smoking status can result in differential misclassification. Future studies that include a mandatory data item regarding smoking status at the time of admission would increase the sensitivity of the variable [32].

## Conclusion

This retrospective cohort study showed that smoking remains a significant yet preventable health burden. Among adults undergoing multiday elective surgery, current smokers had longer hospital LOS, higher rates of readmission and increased costs compared to non-smokers. These results provide an economic justification for hospitals to provide smoking cessation support to patients to reduce health care costs and decrease disease burden alongside other comprehensive measures used to protect adults and children from smoking initiation and tobacco-related harm [12]. Future research that looks into the specific factors contributing to long hospital stays, higher readmission rates and increased costs could help tailor interventions and policies even more effectively to address these situations.

## Data Availability

The datasets analysed during the current study are not publicly available due to a sharing agreement. For further information please contact Dr Gina Arena.
